# Abdominal Surgery

**Published:** 1896-02-15

**Authors:** 


### Progress in Surgery.

### ABDOMINAL SURGERY.

**Topographical Anatomy.**—Thomson[1] thinks the present method of dividing the abdomen into nine regions is unsatisfactory, as in only two of the regions do the descriptive terms directly apply, viz., the umbilical and hypogastric areas. He considers it important that the delineating lines should pass through fixed points which should be readily accessible, and proposes the following delimiting lines, as represented in the accompanying cut: Two horizontal lines are carried across the surface of the abdomen, one on a level with the most dependent part of the tenth costal arch, called the subcostal line; and another, called the interspinous line, connecting the two anterior superior spines of the ilium. Lines are drawn from the centre of the xiphi-sternal articulation to the anterior superior iliac spines on either side. These are called the ilio-sternal lines. In this way a triangle is constructed, the base of which corresponds to the interspinous line, whilst the sides are formed by ilio-sternal lines. A line drawn from the xiphi-sternal articulation to the symphysis pubis subdivides the triangle above described, and further halves the region which lies below the interspinous line. By such an arrangement the surface of the abdomen is marked off into ten regions, to which the following terms might be applied:—

| | | | |
|---|---|---|---|
| Regio hypochondriaca dextra. | Regio epigastrica dextra. | Regio epigastrica sinistra. | Regio hypogastrica sinistra. |
| Regio abdominis lateralis dextra. | Regio umbilicalis dextra. | Regio umbilicalis sinistra. | Regio abdominis lateralis sinistra. |
| | Regio inguinalis dextra. | Regio inguinalis sinistra. | |

It is claimed as an advantage that with little difficulty lines in correspondence with the foregoing can be easily marked off the back of the trunk.

**The Median Incision** usually employed in abdominal operations is, according to Ramsay,[2] a surgical error. The supposed advantages are: (1) Vascularity is low and therefore hæmorrhage is less, but Ramsay thinks it is this absence of vascularity which tends to delay the healing process, and therefore predisposes to hernia. (2) The structures to cut through are fewer and less important, but their similarity renders it difficult, especially to young operators, to know the exact depth of the incision. (3) There is greater facility of access to all parts of the abdominal cavity. This, he thinks, is the only valid argument in favour of a median incision, but considers it more theoretical than practical. The ideal incision for abdominal section is one vertically through the middle of the rectus muscle on either side, for the following reasons: (1) The hæmorrhage is easily controlled, and, as pointed out above, the increased vascularity has advantages as regards the healing process. (2) The injury to the muscle is slight, for after the fascia is divided the muscular fibres are easily separated with a director. (3) If it be necessary to prolong the incision the umbilicus gives rise to no inconvenience. (4) The layers are so well marked that it is impossible with ordinary care to wound the viscera. (5) Access to all parts of the abdominal cavity is just as easy as in the middle line. (6) The scar left looks as if there had been a skin incision only, and the separate layers are not coherent. (7) If the wound is properly closed the risk of hernia is reduced to a minimum. The best method is to close the wound with silkworm gut sutures, running through all the layers. The divided peritoneum, muscle, and fasciæ are brought together separately after the sutures passing through all the layers are in situ.

**Traumatic Lesions of the Abdomen** and its viscera are difficult to diagnose, and Bryant[3] quotes cases to show that abdominal injuries which have been brought about by severe external violence, and are associated with external features and general symptoms which suggest grave internal complications, may in their progress prove that such an inference was incorrect, and that the internal injury was really trivial. Conversely, too, the gravest internal abdominal injuries may co-exist with the slightest or with no external evidence of mischief, and even without shock or collapse. The history of the injury, as showing the amount and mode of application of the force, is important. Thomson[4] considers that in cases of this nature, especially if the violence has been sustained over a limited area, we should first exclude rupture of the bladder, and then search for evidence of internal hæmorrhage or escape of gas. If either of these be suspected, and if the nature of the injury is such as to corroborate the inference from physical examination, the surgeon should without delay make an exploratory laparotomy above or below the umbilicus according to the indications presented. In considering the pros and cons of such an operation it should be remembered that a ruptured abdominal viscus, if treated on the expectant plan, is almost invariably fatal. It is hopeless to delay interference till shock has passed off, for, should a rupture exist, the phenomena of shock will only yield to those of perforative peritonitis. The injection of hot saline fluids into the veins or peritoneal cavity is a valuable procedure in alleviating shock. As regards the necessity of operative procedure, Mumford[5] practically agrees with these views.

**Intestinal Occlusions after Laparotomies** have been specially studied by Adenot,[6] who concludes :—(1) A certain number of post-operative intestinal occlusions have as their cause a physiological occlusion of the colon at the left subcostal angle. This impermeability is favoured by the fibrous character of the suspensory ligament of that angle. It may be also produced by the pressure of the small intestine upon that angle, the acuteness of which is also increased by the downward displacement of the transverse colon. (2) If the small intestine is placed on a plane posterior to the colon after the operation, there is much less danger of its pressing upon this angle and producing the occlusion. (3) Catheterism of the rectum with Fancher's tube may be successful in some cases in reducing the occlusion, either alone or in connection with gases or fluids injected through it. (4) The introduction of the soft tube is not always possible, on account of its doubling up in the cavity of the rectum. The rectal sound should be used with the greatest care, to avoid injuring the mucous membrane. (5) In laparotomies the knuckles of small intestine that lie in the neighbourhood of this particular angle should be displaced, as their presence there threatens to produce compression. (6) It seems preferable to purge the patient gently on the first or by the third day after operation, and not to wait for an exaggerated meteorism, which is of itself a cause of impermeability of the left colic angle. (7) If an occlusion manifests itself after a laparotomy the abdomen should be opened immediately, and, besides the other occlusions examined for, that produced in the left subcostal angle of the colon by the causes mentioned above should not be overlooked. Ziegenspeck[7] says he would be inclined, if obliged to reopen the abdomen on account of ileus, to search for the obstacle in the first place in the true pelvis, on the assumption that it will frequently be found to be connected with the pedicle (after ovariotomy) or operative wound. Strumpf[8] is of opinion that it is useless to reopen the abdomen in cases of paralytic ileus, and that copious rectal injections, nutrient enemata, and medical treatment will prove more beneficial than operation. It is absolutely certain that the employment of asepsis, especially by dry means, materially reduces the number of cases of intestinal obstruction. Tauffer[9] has presented statistics of the cases of ileus which have occurred in his clinique during the antiseptic period, and since he has exclusively used asepsis; during the first period, of 443 operations, ten, or 2·25 per cent., were followed by intestinal obstruction; while during the second period, in 348 operations, he has only had two cases, or ·57 per cent., of this complication.

**A Rare Condition of the Omentum** was found by Smith[10] during an abdominal section for the removal of a solid pelvic tumour. Coils of vessels protruded which exactly, in colour and size, resembled fat, well-developed earth-worms. They were firm, tense, and glistening, and on close examination proved to be the vessels of the omentum. Here and there small tags of areolar tissue containing a little fat could be found on the vessels; but the whole omentum was transformed into a network of large vessels without any fat. A good many vessels were adherent to the tumour; these were tied in a bunch and divided. The rest of the omentum was left intact. Tait has met with a similar condition.

[1] Brit. Med. Jour,, Nov. 30, 1895, p. 1341. [2] Lancet, Nov. 30, 1895. [3] Lancet, Nov. 2, 1895, p. 1097. [4] Edinburgh Hosp. Reports, Vol. II.; and Brit. Med. Jour.. Nov., 9, 1895. [5] Boston Med. and Surg. Jour., July 25, 1895; and Internat. Med. Mag., Oct.. 1895. [6] Gaz. Hebdom. de Med et de Chir., No. 11, 1895, and Internat. Med. Mag., Aug., 1895. [7] Amer. Med. and Surg. Bulletin, p. 7, Sept. 1, 1895. [8] Ibidem. [9] Ibidem. [10] Lancet, Aug. 10, 1895, p. 331.

